# Synthesis of *Giardia* Species and Genotypes in Wild Birds: A Review

**DOI:** 10.3390/vetsci12090911

**Published:** 2025-09-19

**Authors:** Diana Echeverry, Pablo Oyarzún-Ruiz, Carlos Landaeta-Aqueveque

**Affiliations:** 1 Escuela de Medicina Veterinaria, Facultad de Medicina Veterinaria, Universidad San Sebastián, Lientur 1457, Concepcion 4030000, Chile; 2 Laboratorio de Parasitología, Departamento de Microbiología, Facultad de Ciencias Biológicas, Universidad de Concepción, Concepcion 4030000, Chile; poyarzun@udec.cl; 3 Departamento de Patología y Medicina Preventiva, Facultad de Ciencias Veterinarias, Universidad de Concepción, Chillan 4030000, Chile; clandaeta@udec.cl

**Keywords:** avian, assemblages, parasite, protozoan, zoonotic risk

## Abstract

Globally, *Giardia* infections affect an estimated 280 million people each year, making it a significant cause of enteric diarrheal disease. Despite being categorized as a neglected disease, the prevalence of *Giardia* in wild animals varies, with both mammals and birds serving as hosts. Molecular tools have identified nine *Giardia* species, including *Giardia duodenalis* with eight genotypes. Birds, with their wide-ranging habitats and movement capabilities, are crucial disseminators of *Giardia*. This review aimed to identify *Giardia* species in wild birds globally, utilizing various academic databases with search terms such as “wild birds” and “*Giardia*” in combination with Boolean operators for comprehensive results. *Giardia* spp. have been identified in thirteen orders of wild birds; however, they have been genotyped in only seven orders. *Giardia duodenalis* assemblage A was identified in wild birds from the Americas; meanwhile, assemblages A, B, D, and F were identified from Europe, and B and E from Asia. Assemblage B is mainly associated with humans and a wide number of wild animals. Therefore, ascertaining the prevalence of *G. duodenalis* and its assemblages across diverse avian species and orders is crucial to assess the potential risk of zoonotic transmission associated with the dissemination of this protozoan.

## Simple Summary

*Giardia* infections impact over 200 million people yearly, causing significant diarrheal disease. Wild animals, especially mammals and birds, host various *Giardia* types. Molecular tools have identified nine *Giardia* species, notably *Giardia duodenalis*, with eight genotypes. Birds are key *Giardia* carriers that spread the parasite. A global study targeting wild birds was performed to identify *Giardia* species using academic databases and precise search terms. While *Giardia* spans thirteen bird orders, it is genotyped in only seven orders. Different assemblages exist worldwide, with assemblage B linked to humans and many animals. Understanding *G. duodenalis* prevalence and its diverse assemblages in avian species is vital to assess zoonotic risks from the spread of this parasite.

## 1. Introduction

*Giardia* is an anaerobic flagellate protozoan that parasitizes a wide range of vertebrate hosts. *Giardia* species are characterized by their bilateral symmetry and the presence of two symmetrical nuclei in the trophozoites. The taxonomy of *Giardia* species has been debated for many years due to two problems: the first description in 1859 presented confusion with similar names, and later descriptions were based on the type of host rather than the parasite, leading to an excess of described species [1]. Strictly speaking, the first species described was *Giardia duodenalis* (previously known as *intestinalis* and *lamblia*), by Van Leeuwenhoek in 1681, a record that had been lost, and its first description had been attributed to Lamb in 1859 [2]. Subsequently, *Giardia agilis* was described in the intestine of amphibians by Kunstler in 1882 [3]. Historically, *Giardia* species were originally described using the morphology and biological data of the protozoan, and assuming a host specificity of every taxon, the latter of which is now known to be incorrect [3]. Subsequently, this classification was changed with the advance of molecular tools, and to date, nine species have been described within this genus: *G. agilis*; *G. ardeae*; *G. psittaci*; *G. microti*; *G. muris*; *G. varani*; *G. duodenalis*; and, recently, *G. paramelis* and *G. cricetidarum* [4].

*Giardia duodenalis* has eight genotypes according to an analysis of the sequences of several genes; these are known as assemblages, labelled from A to H [5]. Each assemblage consists of a large cluster in the phylogenetic tree, constructed from the sequences of every locus. Some of the assemblages have robust subdivisions in phylogenetic analyses, forming subclusters of well-supported sequences across multiple genetic loci, known as sub-assemblages. For example, within assemblage A, there are three common sub-assemblages, known as AI, AII, and AIII [6]. The genomes of the assemblages have been sequenced in recent years, revealing significant differences between them [7,8,9]. Assemblages A and B can infect non-human animals, as well as humans, with assemblage B being responsible for most infections, and some sub-assemblages present zoonotic potential [10]; AI and AIII are mainly found in animals, while AII is widely detected in humans [4,11]. Assemblage B is mainly associated with humans, livestock, and a wide number of wild animals (mainly mammals and birds), which raises public health concerns [12]. Meanwhile, assemblages C–H show higher host specificity [6]. Furthermore, wild animals can be infected with different *G. duodenalis* assemblages and sub-assemblages, making the determination of the role of wild species in the epidemiology of giardiasis difficult [6,13]. On the other hand, *G. duodenalis* assemblages C, D, E, and F have been sporadically isolated from humans; however, it is suggestive of potential zoonotic transmission [6,10].

**Life Cycle**

The life cycle of *Giardia* is simple, including the proliferating trophozoite and the infective cyst stage [14]. Once the host ingests these cysts, these cysts reach the duodenum, where the release of trophozoites occurs. Then, these trophozoites replicate asexually through binary fission, and some of them differentiate into cysts, to later be disseminated through feces [15]. *Giardia* cysts are environmentally stable as they are tolerant to disinfectants and environmental degradation, and immediately infective [16]. In addition, *Giardia* cysts, which have four nuclei inside, have a low metabolic rate, which allows them to survive up to 12 weeks in cool and moist environments [17,18].

**Diagnosis of *Giardia***

The methods most commonly used for the diagnosis of *Giardia* in feces are light microscopy, immunodiagnostics, and molecular characterization. Until the year 2000, scanning electron microscopy (SEM) was the method of choice for distinguishing between *Giardia* species of mammalian and avian origin. This technique measured the length of the caudal flagellum, as well as symmetry and asymmetry, to differentiate among species. However, additional studies have raised doubts about the reliability of microscopy as a method for this distinction, as sample preparation and the age of trophozoite cultures can result in anomalies in the length of the caudal flagellum [19]. On the other hand, immunoassays continue to be important tools in the diagnosis of giardiasis with high sensitivity and specificity, but they do not provide the option to determine the species of the parasite as Polymerase Chain Reaction (PCR) assays do [3,5].

The molecular characterization of *Giardia* involves initial species classification, followed by genetic identification of their assemblage. To carry out this process, some specific loci serve as markers. The small ribosomal unit RNA (ssuRNA) gene and the elongation factor 1 (ef1a) gene are used to differentiate between species [20]. For assembly and sub-assembly identification, the most common approach is through multiloci genotyping (MLG), performed with the initial amplification of highly conserved genes, such as glutamate dehydrogenase (gdh), triose phosphate isomerase (tpi), and beta giardin (bg) [21]. Likewise, following PCR, restriction enzymes can be employed to assess polymorphisms within the amplified products [6]. The complete genome of the protozoan can also be sequenced from cysts and trophozoites obtained directly from fecal material [22].

**Importance of giardiasis in human, animal, and ecosystem health**

Annually, about 280 million people worldwide suffer from symptomatic *G. duodenalis* infections [16,23]. Although *Giardia* infection is mainly associated with developing countries in Asia, Africa, and Latin America, the National Center for Emerging and Zoonotic Infectious Diseases (2014) reports giardiasis as the third leading cause of diarrheal disease from contaminated water in the United States. According to the Food and Agriculture Organization of the United Nations (FAO) and the World Health Organization (WHO), *Giardia* is the 11th most common cause of morbidity and mortality out of 24 foodborne parasites [24]. The full extent of the public health impact of this parasite is unclear due to variations in reporting and surveillance systems, differences in testing criteria, and different laboratory diagnostic methods [25].

Given that *Giardia* can be transmitted between wildlife and domestic animals and between humans and animals (zoonotic), gaining a deeper understanding of the present distribution patterns and possible transmission routes among wildlife is crucial for the health of humans, domestic animals, and wildlife alike [26].

In addition to the above considerations, the wide host range of *Giardia* plays an important role in the environmental dissemination of the parasite, contaminating food and water sources, contributing to maintaining the life cycle of the protozoan [6,10,21,27]. Two species of *Giardia* are responsible for causing avian giardiasis: *G. ardeae* and *G. psittaci* [10]. In addition to these two species, *G. duodenalis* has also been reported in wild birds, including the zoonotic assemblages A and B, as well as non-zoonotic assemblages D and F, and to be potentially zoonotic as assemblage E [12,28,29].

Birds have certain traits that facilitate the spread of pathogens, which could be more noticeable in birds adapted to anthropized landscapes. Some of these traits are the ability to travel long distances and inhabit aquatic and/or terrestrial ecosystems, and some birds may come into contact with human waste, for example, in landfills or household garbage; transit through areas near animal production farms; and be in environments highly contaminated by waste from other animal species, for example, wetlands and green areas contaminated with canine and feline feces [30]. Additionally, the transmission cycle can be maintained among wild species inhabiting the same ecosystem, promoting the parasite’s persistence in the environment and in hosts [31].

*Giardia* is a waterborne disease for which infection rates and distribution are projected to increase in response to global climate change scenarios [30]. Therefore, this protozoan has been identified as a priority for One Health, as it is a zoonotic disease and little is known about its prevalence in wild animals [32].

**Effects of Climate Change on the Distribution of *Giardia* and its Transmission**

Several studies have focused on evaluating the prevalence, risk factors, and variables that influence the global occurrence of parasites such as *Giardia* [33]. Climate change can trigger ecological disruptions that alter the transmission mechanisms of pathogens. This phenomenon, known as the intersection of climate change with transmission dynamics or ecological fitting, may enable the emergence of parasites and diseases without requiring evolutionary changes in their capacity to utilize hosts [34]. This implies that parasites may colonize new hosts or increase prevalence, morbidity, and mortality in already known hosts. Climate change also promotes the movement and migration of wild species, carrying with them the pathogenic organisms that infect them [35]. For example, the northward expansion of *Giardia* from subarctic to Arctic environments has been documented in a variety of migratory and resident species [36,37].

Several studies have reported positive associations between environmental variables—many linked to the hydrological cycle, such as increased precipitation and temperature, permafrost melting, and coastal flooding—and the occurrence of giardiasis in humans and animals [31,38]. However, these variables may either promote the presence of *Giardia* or, conversely, reduce its prevalence depending on geographic location [31]. For instance, one study documented a consistent seasonal rise in giardiasis cases in temperate, developed countries during the summer months, possibly because higher temperatures prolong the persistence of cysts and oocysts in the environment [39,40]. Evidence suggests that the protozoan pathogen *Giardia* is particularly sensitive to environmental changes due to its environmentally mediated life cycle [39]. Consequently, further studies are needed to support epidemiological surveillance of this parasite in the context of climate change, as the full impacts of these changes—especially under extreme events—remain difficult to predict or project [38].

Birds play a key role as carriers of *Giardia* and other parasitic diseases, and their interaction with climate change is a major concern. Rising temperatures and the expansion of geographic areas favorable to the survival and transmission of *Giardia* may allow migratory birds to transport and disperse the parasite into new regions. Moreover, shifts in bird migration and movement in response to climate change could enhance the spread of *Giardia* along different migratory routes. These changes in bird migration patterns and distributions, combined with climate-induced ecosystem disruptions, may significantly influence the prevalence and incidence of *Giardia* across regions. This highlights the urgent need to investigate and monitor the interplay between *Giardia*-carrying birds and climate change in order to mitigate risks to both public and environmental health.

The aim of this literature review was to identify the presence of *Giardia* species and assemblages among various orders of wild birds worldwide, drawing upon the published scientific literature.

## 2. Materials and Methods

A comprehensive literature review was conducted from original scientific articles and case reports spanning the period from 1990 to March of 2024. This review focused on extracting information not only on the species of parasitized wild birds but also on the geographic regions or areas where *Giardia* occurrences have been documented.

A bibliographic search was performed using the following search engines and information sources: Google Scholar, ProQuest, PubMed, ScienceDirect, and Scopus. The search was performed using the keywords *wild birds*, *Giardia*, *assemblage*, *avian*, and *birds*, with the Boolean operators ‘’AND’’ and “OR”. Only scientific papers published in English were considered. Among these publications, original articles, short communications, and case reports were included. Reports of *Giardia* in domestic birds and other domestic or wild animals were not considered. Regardless of the article type, the selected references were given equal weight, provided they met the established inclusion and exclusion criteria.

**Figure 1:**
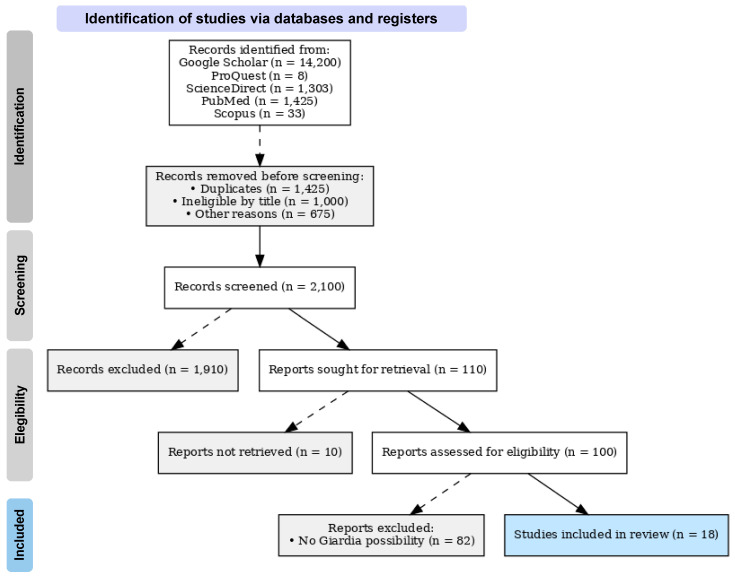
Prisma flow diagram of the papers selected for this review. This diagram explains how the search was conducted and the academic articles selected for the review according to the inclusion and exclusion criteria.

A first search with the keywords without Boolean operators yielded a total of 14,200 results. Then, a search was performed using the Boolean operators separately with the keywords. The titles of the first 100 scientific articles were reviewed, and those unrelated to the research interests were excluded. Subsequently, selection was made by reading the abstract to determine whether the study presented results on *Giardia* positivity in wild birds (Figure 1).

To complete the bibliographic search, the Connected Papers tool (https://www.connectedpapers.com/ (accessed on 10 September 2024)) was used to identify the most important authors related to the topic of interest and to refine the bibliographic search. In this search, 41 bibliographic references were identified, which were also reviewed considering the inclusion and exclusion criteria (Figure S1). After the screening, 7 references were considered, which coincide with the initial search. The list of references identified by Connected Papers can be found in the Supplementary Materials.

In this search, the study by Reboredo-Fernandez et al. [28] was used as the base reference, since the publication contained more information on *Giardia* in wild birds (Figure S1). From this reference, a cluster map was constructed, showing the relationships between the main authors on the topic of interest (https://www.connectedpapers.com/main/45bf46e5aad407c2d5ca9a22cea672b863ccaa66/graph?utm_source=share_popup&utm_medium=email&utm_campaign=share_graph (accessed on 17 June 2025)).

The search was filtered, and inconclusive studies on *Giardia* positivity were excluded. Finally, 18 scientific articles provided data related to the variables of interest.

The variables analyzed to achieve the general objectives of this study were as follows: order, bird species, *Giardia* species or assemblage, country, year, and author. A descriptive analysis was performed to summarize the information regarding the variables. The graph presented in this article illustrates the distribution of reports by geographic area (country), along with the predominant avian order represented in each area.

## 3. Results

**Figure 2:**
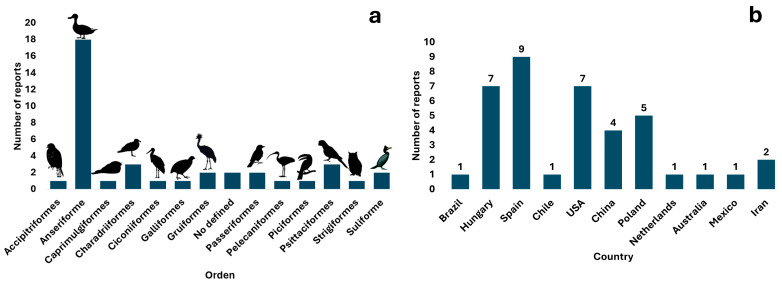
Graphical representation of the number of reports identified by order of wild birds (**a**) and by country (**b**).

The analysis of the scientific literature resulted in the identification of *Giardia* spp. in 32 species and 13 orders of wild birds. According to the published literature, birds belonging to the order Anseriformes are the most frequently reported hosts worldwide (seven reports in six countries) and have the highest number of infected species (twelve), followed by Charadriiformes and Psittaciformes (two reports and three host species each (Figure 2a, Table 1).

**Figure 3:**
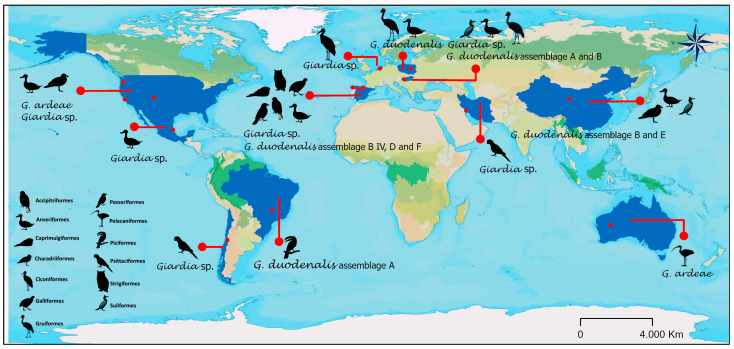
Global distribution of *Giardia* species and genotypes of *Giardia duodenalis* in wild birds.

These reports were found mainly in Europe (twenty-one reports) (six references and seventeen host species) and North America (eight reports) (four references and eight host species). In the case of *G. duodenalis* and its genotyping, assemblage A is reported in America (Brazil); assemblages A, B, D, and F in Europe (Spain and Hungary); and assemblages B and E in Asia (China). Spain has the most orders of birds carrying identified *G. duodenalis* (four orders: Anseriformes, Accipitriformes, Galliformes, and Passeriformes), from which *G. duodenalis* has been genotyped, followed by China (three orders: Anseriformes, Charadriiformes, and Suliformes) (Figure 2b and Figure 3). According to the literature search and bibliometric analysis, the first genotyping study of *G. duodenalis* was conducted by Plutzer and Tomo in 2009 (see Supplementary Figure S1). A list of published studies on *Giardia* in wild birds is provided in Table 1. Two studies reported the presence of *Giardia* in waterfowl feces but did not define the species of birds that were carriers or the orders to which they belong: Cano et al. [30] identified *G. duodenalis* assemblage B IV, and Shemshadi et al. [51] reported *G. duodenalis*. A list of published studies on *Giardia* in wild birds is provided in Table 1.

## 4. Discussion

This study offers an analysis of the global presence of *Giardia* in wild birds, delineating the species and orders harboring the protozoan and highlighting studies where genotyping of *G. duodenalis* has been accomplished. Birds serve as sentinels, and detecting pathogens within this taxon holds significant importance for epidemiological surveillance [32]. For *G. ardeae*, the findings are limited to two bird species, with only two studies reported up to the search date [42,45]. However, these studies date back to the nineties, and recent studies have not reported the presence of *G. ardeae*. This may be due to the fact that initially, *Giardia* species were differentiated microscopically based on the difference in the length of the caudal flagellum. However, subsequent studies have confirmed that this is not an appropriate technique for discriminating between *Giardia* species [19,42].

On the other hand, wild birds harboring *Giardia* spp. have been reported in Asia, Europe, North America, South America, and Oceania, with highly variable prevalences, ranging from 3% to 49% [26,32,33,34,35,36,37,38,39,40,41,42,43,44,45,46,47,48,49,50,51,52]. Most bird orders reported as carriers of *Giardia* share the commonality that they are marine or freshwater species. Several studies agree that both wild and captive birds carrying this protozoan are waterfowl [26,32,44,50]. This finding coincides with those of previous reports indicating the contamination of surface water with pathogens probably of avian origin, such as *Giardia*, that have been found in the water source [52].

Prevalences vary by continent, but it is important to consider that these data are specific to some countries and primarily focused on birds of the Anseriformes order. However, *Giardia* has also been identified in birds of other orders and families, which may be exposed to carrying the parasite. In this context, further studies are needed to gather evidence of *Giardia* presence in other bird species, establish prevalences, and assess risk factors that may be associated with *Giardia* presence in avian species.

Wild birds share their habitat with other domestic or exotic bird species and mammals, which could potentially act as hosts for *Giardia* [53,54]. In the case of invasive birds, such as pigeons, which have a wide worldwide distribution and may be free-ranging or kept as pets, the prevalences of *Giardia* spp. could reach up to 52% [55]. Unfortunately, not all reports identify the *Giardia* species present in pigeons, but one study identified *G. duodenalis* assemblage E, which is considered the most representative genotype among ruminants and has a zoonotic potential. Additionally, this assemblage has been identified in other wild birds in the Anseriformes, Charadriiformes, and Suliformes orders [56,57]. However, studies genotyping *Giardia* in birds to determine if other potentially zoonotic assemblages are present or to establish which assemblage may be dominant in birds are lacking [58].

To date, molecular studies support the zoonotic potential of some *G. duodenalis* assemblages, but further studies are required to understand the infection dynamics of the protozoan in different animal species and humans. As highlighted in this literature review, most genotyping studies have been conducted in the European continent, leaving a gap in our understanding of what may be occurring in continents such as Africa and the Americas, particularly given the abundance and richness of bird species. For example, in South America, all reported cases are restricted to Brazil (*G. duodenalis* assemblage A) [27]. It highlights the need for research to explore how various species act as reservoirs for *G. duodenalis*, considering human, animal, and ecosystem health. This research would help clarify species interactions and the impact of human activities on the protozoan’s ecology and suggest ways to prevent and control related diseases [59,60]. A recent study that analyzed reports of giardiasis in Latin America concluded that it still represents a neglected public health problem [61].

Wildlife, such as wild birds, can act as hosts for these pathogens, aiding in their transmission among themselves, humans, and the environment, underscoring the complex One Health issue presented by these zoonotic agents [61]. Efforts should focus on improving water, sanitation, and hygiene practices to reduce disease transmission. Additionally, addressing the impact of climatic variability, land-use changes, and social factors in giardiasis is crucial, emphasizing the need for global public health interventions to prevent future disease burden, particularly in regions with limited resources [41]. Therefore, adopting a One Health approach is essential for addressing zoonotic diseases and mitigating the challenges posed by this pathogen.

## 5. Conclusions

In this literature review, we gathered data on the presence of *Giardia* and its assemblages across various orders of wild birds worldwide. While *Giardia* has predominantly been identified in Anseriformes, molecular characterization has largely been restricted to birds from European countries. Therefore, an investigation of the circulation of *Giardia* assemblages and an assessment of whether birds could serve as potential disseminators of this protozoan, as well as the implications for zoonotic risk, are essential.

## Figures and Tables

**Table 1:** Information obtained from the bibliographic search ordered by the variables under study.

Continent	Country	Order	Bird Species	Common Name	Giardia Assemblage	Year	Ref.	Frequency (%)	Prevalence
Asia	China	Anseriformes	*Anser indicus*	Bar-headed goose	*G. duodenalis* assemblages B and E	2021	[12]	3.39 (23/679) *	4.4%
	China	Charadriiformes	*Chroicocephalus brunnicephalus*	Brown-headed Gull	*G. duodenalis* assemblages B and E	2021	[12]	3.39 (23/679) *	
	China	Charadriiformes	*Larus ichthyaetus*	Great black-headed gull	*G. duodenalis* assemblages B and E	2021	[12]	3.39 (23/679) *	
	China	Suliformes	*Phalacrocorax carbo*	Great Cormorant	*G. duodenalis* assemblages B and E	2021	[12]	3.39 (23/679) *	
	Iran	Psittaciformes	*Melopsittacus undulatus*	Common parakeet	*Giardia* sp.	2023	[41]	21.73 (5/23)	
	Iran	Psittaciformes	*Nymphicus hollandicus*	Cockatiel	*Giardia* sp.	2023	[41]	10 (3/30)	
Oceania	Australia	Pelecaniformes	*Threskiornis spinicollis*	Straw-necked ibis	*G. ardeae*	1996	[42]	70 (44/63)	70%
North America	Mexico	Anseriformes	*Anser anser*	Greylag goose	*Giardia* spp.	2023	[43]	100 (2/2)	10%
	USA	Anseriformes	*Ana america*	American wigeon	*Giardia* sp.	2002	[44]	66.7 (2/3)	
	USA	Anseriformes	*Anas acuta*	Northern pintail	Giardia sp.	2002	[44]	100 (1/1)	
	USA	Anseriformes	*Anas discors*	Blue-winged teal	*Giardia* sp.	2002	[44]	25 (1/4)	
	USA	Anseriformes	*Anas platyrhynchos*	Mallard	*Giardia* sp.	2002	[44]	25.5 (13/51)	
	USA	Anseriformes	*Ardea herodias*	Great blue heron	*G. ardeae*	1990	[45]	12.5 (1/8)	
	USA	Anseriformes	*Mergus merganser*	Common merganser	*Giardia* sp.	2002	[44]	33.3 (1/3)	
	USA	Charadriiformes	*Larus* spp.	Gulls	*Giardia* sp.	2012	[46]	2.1 (1/145)	
South America	Brazil	Piciformes	*Ramphastos toco*	Giant toucan	*G. duodenalis* assemblage A	2017	[29]	100 (1/1)	12.4%
	Chile	Psittaciformes	*Myopsitta monachus*	Monk parakeet	*Giardia* sp.	2021	[47]	12 (25/207)	
Europe	Hungary	Anseriformes	*Anas strepera*	Gadwall	*G. duodenalis* assemblage A	2009	[48]	25 (1/4)	13.7%
	Hungary	Anseriformes	*Anser anser*	Greylag goose	*G. duodenalis* assemblage B	2009	[48]	2 (1/48)	
	Hungary	Anseriformes	*Anser fabalis*	Taiga bean	*Giardia* sp.	2009	[48]	12.5 (1/8)	
	Hungary	Anseriformes	*Mareca strepera*	The gadwall	*G. duodenalis* assemblage A	2009	[48]	25 (1/4)	
	Hungary	Gruiformes	*Fulica atra*	Eurasian coot	*Giardia* sp.	2009	[48]	25 (1/4)	
	Hungary	Suliformes	*Phalacrocorax carbo*	Great Cormorant	*Giardia* sp.	2009	[48]	100 (1/1)	
	Netherlands	Ciconiiformes	*Ciconia ciconia*	White stork	*Giardia* sp.	2000	[49]	100 (1/1)	
	Poland	Anseriformes	*Anas platyrhynchos*	Mallard	*G. duodenalis*	2009	[50]	21.9 (7/32)	
	Poland	Anseriformes	*Anser anser*	Greylag goose	*G. duodenalis*	2009	[50]	29.4 (10/34)	
	Poland	Anseriformes	*Cygnus olor*	Mute swan	*G. duodenalis*	2009	[50]	12.5 (4/33)	
	Poland	Anseriformes	*Mergus merganser*	Common merganser	*G. duodenalis*	2009	[50]	1.4 (1/72)	
	Poland	Gruiformes	*Balearica pavonina*	Black crowned crane	*G. duodenalis*	2009	[50]	25 (1/4)	
	Spain	Accipitriformes	*Buteo buteo*	Common buzzard	*G. duodenalis* assemblage B	2015	[28]	1.2 (1/84)	
	Spain	Anseriformes	*Anas platyrhynchos*	Mallard	*G. duodenalis* Assemblage F	2015	[28]	50 (2/4)	
	Spain	Caprimulgiformes	*Caprimulgus europaeus*	European nightjar	*Giardia* sp.	2015	[28]	16.7(1/6)	
	Spain	Galliformes	*Coturnix coturnix*	Common quail	*G. duodenalis* assemblage B	2015	[28]	100 (1/1)	
	Spain	No defined	Waterfowl	Not defined	*G. duodenalis*	2014	[51]	24.2 (71/293)	
	Spain	No defined	Waterfowl	Not defined	*G. duodenalis* assemblage B IV	2016	[30]	8.3 (22/265)	
	Spain	Passeriformes	*Garrulus glandarius*	Eurasian jay	*G. duodenalis* assemblage D	2015	[28]	100 (1/1)	
	Spain	Passeriformes	*Pica pica*	Eurasian magpie	*G. duodenalis* assemblage B	2015	[28]	20 (1/5)	
	Spain	Strigiformes	*Tyto alba*	Barn Owl	*Giardia* sp.	2015	[28]	2 (1/48)	

* In this study, prevalence by bird species is not differentiated; instead, general prevalence is provided. The methodology does not exactly describe how many samples were taken per bird order or species.

## Data Availability

The original contributions presented in this study are included in the article/Supplementary Materials. Further inquiries can be directed to the corresponding author.

## Supplementary Materials

The following supporting information can be downloaded at https://www.mdpi.com/article/10.3390/vetsci12090911/s1, Figure S1: Graphical summary of the main authors reporting on *Giardia* and *Giardia* species in birds, created using the Connected Papers tool. Supplementary reference: Include all reference from Connected Papers search. Supplementary references [62,63,64,65,66,67,68,69,70,71,72,73,74,75,76,77,78,79,80,81,82,83,84,85,86,87,88,89,90,91,92,93,94].
