# Supplementary material for: Synthesis of Giardia Species and Genotypes in Wild Birds: A Review
Source: Vet Sci. 2025 Sep 19;12(9):911. doi: 10.3390/vetsci12090911 (PMC12474223; doi:10.3390/vetsci12090911)
Supplement: Supplementary file 1 [file vetsci-12-00911-s001.zip › vetsci-3819779-Supplementary Figure S1.pdf]

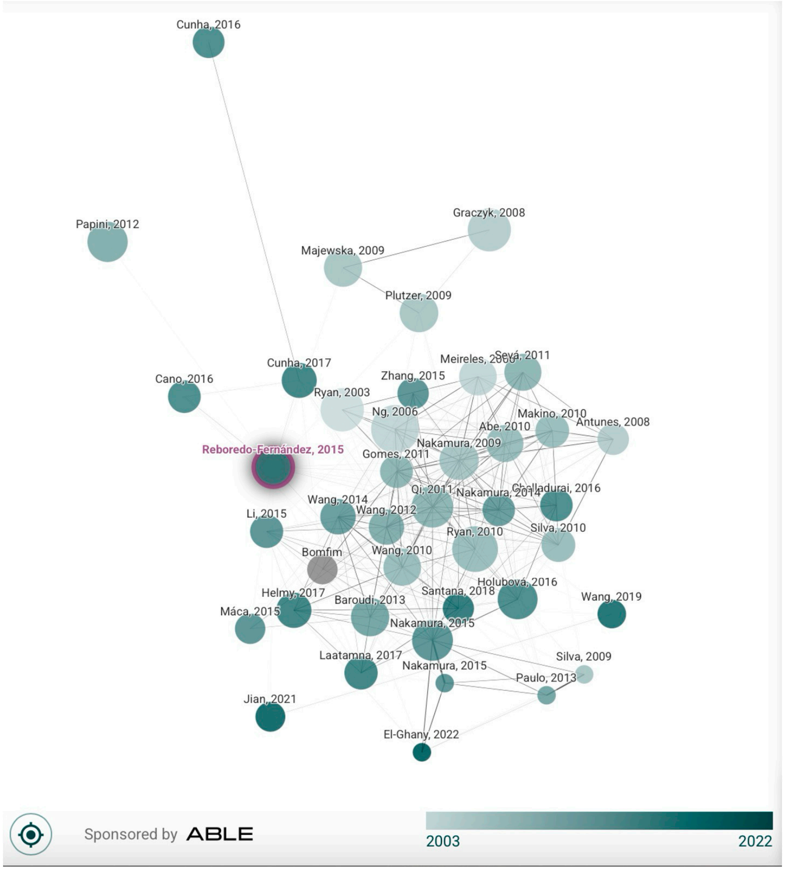

Figure S1. Graphical summary of the main authors reporting on *Giardia* and *Giardia* species in birds, created using the Connected Papers tool.
