# Supplementary material for: Synthesis of Giardia Species and Genotypes in Wild Birds: A Review
Source: Vet Sci. 2025 Sep 19;12(9):911. doi: 10.3390/vetsci12090911 (PMC12474223; doi:10.3390/vetsci12090911)
Supplement: Supplementary file 1 [file vetsci-12-00911-s001.zip › vetsci-3819779-Supplementary References.pdf]

References

1. Reboredo-Fernandez, A.; Ares-Mazás, E.; Caccio, S.M.; Gómez-Couso, H. Occurrence of *Giardia* and *Cryptosporidium* in Wild Birds in Galicia (Northwest Spain). *Parasitology* **2015**, *142*, 917–925. https://doi.org/10.1017/S0031182015000049.
2. Jian, Y.; Zhang, X.; Li, X.; Schou, C.; Charalambidou, I.; Ma, L.; Karanis, P. Occurrence of *Cryptosporidium* and *Giardia* in Wild Birds from Qinghai Lake on the Qinghai-Tibetan Plateau, China. *Parasitol. Res.* **2021**, *120*, 615–628. https://doi.org/10.1007/s00436-020-06993-w.
3. Plutzer, J.; Tomor, B. The Role of Aquatic Birds in the Environmental Dissemination of Human Pathogenic *Giardia duodenalis* Cysts and *Cryptosporidium* Oocysts in Hungary. *Parasitol. Int.* **2009**, *58*, 227–231. https://doi.org/10.1016/j.parint.2009.05.004.
4. Majewska, A.C.; Graczyk, T.K.; Słodkowicz-Kowalska, A.; Tamang, L.; Jędrzejewski, S.; Zduniak, P.; Solarczyk, P.; Nowosad, A.; Nowosad, P. The Role of Free-Ranging, Captive, and Domestic Birds of Western Poland in Environmental Contamination with *Cryptosporidium parvum* Oocysts and *Giardia lamblia* Cysts. *Parasitol. Res.* **2009**, *104*, 1093–1099. https://doi.org/10.1007/s00436-008-1293-9.
5. Papini, R.; Girivetto, M.; Marangi, M.; Mancianti, F.; Giangaspero, A. Endoparasite Infections in Pet and Zoo Birds in Italy. *Sci. World J.* **2012**, *2012*, 253127. https://doi.org/10.1100/2012/253127.
6. Cano, L.; de Lucio, A.; Bailo, B.; Cardona, G.A.; Muadica, A.S.O.; Lobo, L.; Carmena, D. Identification and Genotyping of *Giardia* Spp. and Cryptosporidium Spp. Isolates in Aquatic Birds in the Salburua Wetlands, Álava, Northern Spain. *Vet. Parasitol.* **2016**, *221*, 144–148. https://doi.org/10.1016/j.vetpar.2016.03.026.
7. da Cunha, M.J.R.; Cury, M.C.; Santín, M. Molecular Identification of *Enterocytozoon Bieneusi*, *Cryptosporidium*, and *Giardia* in Brazilian Captive Birds. *Parasitol. Res.* **2017**, *116*, 487–493. https://doi.org/10.1007/s00436-016-5309-6.
8. Bomfim, T.C.B.; Gomes, R.S.; Huber, F.; Couto, M.C.M. The importance of poultry in environmental dissemination of Cryptosporidium spp. *Open Vet. Sci. J.* **2013**, *7*, 12–17. https://doi.org/10.2174/1874318801307010012
9. Qi, M.; Wang, R.; Ning, C.; Li, X.; Zhang, L.; Jian, F.; Sun, Y.; Xiao, L. *Cryptosporidium* spp. in pet birds: genetic diversity and potential public health significance. *Exp. Parasitol.* **2011**, *128*, 336–340. https://doi.org/10.1016/j.exppara.2011.04.003
10. Wang, R.; Wang, F.; Zhao, J.; Qi, M.; Ning, C.; Zhang, L.; Xiao, L. *Cryptosporidium* spp. in quails (*Coturnix coturnix japonic*a) in Henan, China: molecular characterization and public health significance. *Vet. Parasitol.* **2012**, *187*, 534–537. https://doi.org/10.1016/j.vetpar.2012.02.002
11. Nakamura, A.A.; Simões, D.; Antunes, R.; Silva, D.C.D.; Meireles, M. Molecular characterization of *Cryptosporidium* spp. from fecal samples of birds kept in captivity in Brazil. *Vet. Parasitol.* **2009**, *166*, 47–51. https://doi.org/10.1016/j.vetpar.2009.07.033
12. Antunes, R.; Simões, D.; Nakamura, A.A.; Meireles, M. Natural Infection with Cryptosporidium galli in Canaries (Serinus canaria), in a Cockatiel (Nymphicus hollandicus), and in Lesser Seed-Finches (Oryzoborus angolensis) from Brazil. *Avian Dis.* **2008**, *52*, 702–705. https://doi.org/10.1637/8356-051208-Case.1
13. Nakamura, A.A.; Homem, C.G.; Silva, A.J.D.D.; Meireles, M. Diagnosis of gastric cryptosporidiosis in birds using a duplex real-time PCR assay. *Vet. Parasitol.* **2014**, *205*, 7–13. https://doi.org/10.1016/j.vetpar.2014.07.033
14. Li, J.; Lin, X.; Zhang, L.; Qi, N.; Liao, S.; Lv, M.; Wu, C.; Sun, M. Molecular characterization of *Cryptosporidium* spp. in domestic pigeons (*Columba livia domestica*) in Guangdong Province, Southern China. *Parasitol. Res.* **2015**, *114*, 2237–2241. https://doi.org/10.1007/s00436-015-4415-1
15. Nakamura, A.A.; Meireles, M. *Cryptosporidium* infections in birds-a review Infecção por Cryptosporidium em aves-uma revisão. *Rev. Bras. Parasitol. Veterinária* **2015**, *24*, 253–267.
16. Santana, B.N.; Kurahara, B.; Nakamura, A.A.; Camargo, V.D.S.; Ferrari, E.D.; Silva, G.S.D.; Nagata, W.; Meireles, M. Detection and characterization of Cryptosporidium species and genotypes in three chicken production systems in Brazil using different molecular diagnosis protocols. *Prev. Vet. Med.* **2018**, *151*, 73–78. https://doi.org/10.1016/j.prevetmed.2018.01.007
17. Sevá, A.P.; Funada, M.R.; Richtzenhain, L.; Guimarães, M.B.; Souza, S.; Allegretti, L.; Sinhorini, J.; Duarte, V.V.; Soares, R. Genotyping of Cryptosporidium spp. from free-living wild birds from Brazil. *Vet. Parasitol.* **2011**, *175*, 27–32. https://doi.org/10.1016/j.vetpar.2010.09.031
18. Baroudi, D.; Khelef, D.; Goucem, R.; Adjou, K.; Adamu, H.; Zhang, H.W.; Xiao, L. Common occurrence of zoonotic pathogen Cryptosporidium meleagridis in broiler chickens and turkeys in Algeria. *Vet. Parasitol.* **2013**, *196*, 334–340. https://doi.org/10.1016/j.vetpar.2013.02.022
19. Ryan, U. Cryptosporidium in birds, fish and amphibians. *Exp. Parasitol.* **2010**, *124*, 113–120. https://doi.org/10.1016/j.exppara.2009.02.002

20. Cunha, M.J.D.D.; Cury, M.; Santín, M. Widespread presence of human-pathogenic Enterocytozoon bieneusi genotypes in chickens. *Vet. Parasitol.* **2016**, *217*, 108–112. https://doi.org/10.1016/j.vetpar.2015.12.019
21. Makino, I.; Abe, N.; Reavill, D. Cryptosporidium Avian Genotype III as a Possible Causative Agent of Chronic Vomiting in Peach-Faced Lovebirds (Agapornis roseicollis). *Avian Dis.* **2010**, *54*, 1102–1107. https://doi.org/10.1637/9227-123009-Case.1
22. Abe, N.; Makino, I. Multilocus genotypic analysis of Cryptosporidium isolates from cockatiels, Japan. *Parasitol. Res.* **2010**, *106*, 1491–1497. https://doi.org/10.1007/s00436-010-1810-5
23. Ryan, U.; Xiao, L.; Read, C.; Sulaiman, I.; Monis, P.; Lal, A.; Fayer, R.; Pavlásek, I. A Redescription of *Cryptosporidium galli* pavlasek, 1999 (apicomplexa: cryptosporidiidae) from birds. *J. Parasitol.* **2003**, *89*, 809–813. https://doi.org/10.1645/GE-74RI
24. Holubová, N.; Sak, B.; Hořčičková, M.; Hlásková, L.; Květoňová, D.; Menchaca, S.; Mcevoy, J.; Kváč, M. Cryptosporidium avium n. sp. (Apicomplexa: Cryptosporidiidae) in birds. *Parasitol. Res.* **2016**, *115*, 2243–2251. https://doi.org/10.1007/s00436-016-4967-8
25. Graczyk, T.; Majewska, A.; Schwab, K. The role of birds in dissemination of human waterborne enteropathogens. *Trends Parasitol.* **2008**, *24*, 55–59. https://doi.org/10.1016/j.pt.2007.10.007
26. Wang, L.; Xue, X.; Li, J.Q.; Zhou, Q.J.; Yu, Y.; Du, A. Cryptosporidiosis in broiler chickens in Zhejiang Province, China: molecular characterization of oocysts detected in fecal samples. *Parasite* **2014**, *21*, 36. https://doi.org/10.1051/parasite/2014035
27. Wang, R.; Jian, F.; Sun, Y.; Hu, Q.; Zhu, J.; Wang, F.; Ning, C.; Zhang, L.; Xiao, L. Large-scale survey of Cryptosporidium spp. in chickens and Pekin ducks (Anas platyrhynchos) in Henan, China: prevalence and molecular characterization. *Avian Pathol.* **2010**, *39*, 447–451. https://doi.org/10.1080/03079457.2010.518314
28. Chelladurai, J.J.; Clark, M.; Kváč, M.; Holubová, N.; Khan, E.; Stenger, B.L.S.; Giddings, C.; Mcevoy, J. Cryptosporidium galli and novel Cryptosporidium avian genotype VI in North American red-winged blackbirds (Agelaius phoeniceus). *Parasitol. Res.* **2016**, *115*, 1901–1906. https://doi.org/10.1007/s00436-016-4930-8
29. Zhang, X.X.; Zhang, N.; Zhao, G.H.; Zhao, Q.; Zhu, X.Q. Prevalence and Genotyping of Cryptosporidium Infection in Pet Parrots in North China. *BioMed Res. Int.* **2015**, *2015*, 549798. https://doi.org/10.1155/2015/549798
30. Silva, D.C. Avaliação física, epidemiológica e molecular da infecção por Cryptosporidium spp. em passeriformes. Master's Thesis, Universidade Estadual Paulista, Sao Paulo, Brazil, 2009.
31. El-Ghany, W.A.A. Avian Cryptosporidiosis: A significant parasitic disease of public health hazard. *Slov. Vet. Res.* **2022**, *59*. https://doi.org/10.26873/svr-1354-2022
32. Meireles, M.; Soares, R.; Santos, M.M.A.B.D.; Gennari, S. Biological studies and molecular characterization of a *Cryptosporidium* isolate from ostriches (*Struthio camelus*). *J. Parasitol.* **2006**, *92*, 623–626. https://doi.org/10.1645/0022-3395(2006)92[623:BSAMCO]2.0.CO;2
33. Wang, K.; Gazizova, A.; Wang, Y.; Zhang, K.; Zhang, Y.; Chang, Y.; Cui, Y.; Zhang, Y.; Zhang, S.M.; Zhang, L. First Detection of Cryptosporidium spp. in Migratory Whooper Swans (*Cygnus cygnus*) in China. *Microorganisms* **2019**, *8*, 6. https://doi.org/10.3390/microorganisms8010006
34. Paulo, S. Determinação da ocorrência de Cryptosporidium galli em amostras fecais de aves por meio da PCR em tempo real. Doctoral Dissertation, Universidade de São Paulo, São Paulo, Brazil, 2013.
35. Laatamna, A.E.; Holubová, N.; Sak, B.; Kváč, M. Cryptosporidium meleagridis and C. baileyi (Apicomplexa) in domestic and wild birds in Algeria. *Folia Parasitol.* **2017**, *64*, 018. https://doi.org/10.14411/fp.2017.018
36. Silva, D.C.; Homem, C.G.; Nakamura, A.A.; Teixeira, W.; Perri, S.; Meireles, M. Physical, epidemiological, and molecular evaluation of infection by Cryptosporidium galli in Passeriformes. *Parasitol. Res.* **2010**, *107*, 271–277. https://doi.org/10.1007/s00436-010-1858-2
37. Helmy, Y.A.; Krücken, J.; Abdelwhab, E.S.M.; Samson-Himmelstjerna, G.V.; Hafez, H. Molecular diagnosis and characterization of Cryptosporidium spp. in turkeys and chickens in Germany reveals evidence for previously undetected parasite species. *PLoS ONE* **2017**, *12*, e0177150. https://doi.org/10.1371/journal.pone.0177150
38. Ng, J.; Pavlásek, I.; Ryan, U. Identification of Novel Cryptosporidium Genotypes from Avian Hosts. *Appl. Environ. Microbiol.* **2006**, *72*, 7548–7553. https://doi.org/10.1128/AEM.01352-06
39. Máca, O.; Pavlásek, I. First finding of spontaneous infections with Cryptosporidium baileyi and C. meleagridis in the red-legged partridge Alectoris rufa from an aviary in the Czech Republic. *Vet. Parasitol.* **2015**, *209*, 164–168. https://doi.org/10.1016/j.vetpar.2015.03.003
40. Gomes, R.S.; Huber, F.; Silva, S.; Bomfim, T.C.B. Cryptosporidium spp. parasitize exotic birds that are commercialized in markets, commercial aviaries, and pet shops. *Parasitol. Res.* **2011**, *110*, 1363–1370. https://doi.org/10.1007/s00436-011-2636-5
